# The Complex Structure of Protein AaLpxC from *Aquifex aeolicus* with ACHN-975 Molecule Suggests an Inhibitory Mechanism at Atomic-Level against Gram-Negative Bacteria

**DOI:** 10.3390/molecules26051451

**Published:** 2021-03-07

**Authors:** Shuai Fan, Danyang Li, Maocai Yan, Xiao Feng, Guangxin Lv, Guangteng Wu, Yuanyuan Jin, Yucheng Wang, Zhaoyong Yang

**Affiliations:** 1 Institute of Medicinal Biotechnology, Chinese Academy of Medical Sciences & Peking Union Medical College, Beijing 100050, China; sfan@imb.pumc.edu.cn (S.F.); lidanyangg@foxmail.com (D.L.); fengxiao931209@163.com (X.F.); guangxinlv@163.com (G.L.); jinyuanyuan@imb.pumc.edu.cn (Y.J.); 2 School of Pharmacy, Jining Medical University, Rizhao, Shandong 276800, China; yanmaocai@126.com; 3 ArNuXon Pharm-Sci Co., Ltd., Beijing 100085, China; wugtom@aliyun.com

**Keywords:** LpxC, ACHN-975, X-ray crystallography, crystal structure ITC, drug design

## Abstract

New drugs with novel antibacterial targets for Gram-negative bacterial pathogens are desperately needed. The protein LpxC is a vital enzyme for the biosynthesis of lipid A, an outer membrane component of Gram-negative bacterial pathogens. The ACHN-975 molecule has high enzymatic inhibitory capacity against the infectious diseases, which are caused by multidrug-resistant bacteria, but clinical research was halted because of its inflammatory response in previous studies. In this work, the structure of the recombinant UDP-3-*O*-(R-3-hydroxymyristol)-*N*-acetylglucosamine deacetylase from *Aquifex aeolicus* in complex with ACHN-975 was determined to a resolution at 1.21 Å. According to the solved complex structure, ACHN-975 was docked into the AaLpxC’s active site, which occupied the site of AaLpxC substrate. Hydroxamate group of ACHN-975 forms five-valenced coordination with resides His74, His226, Asp230, and the long chain part of ACHN-975 containing the rigid alkynyl groups docked in further to interact with the hydrophobic area of AaLpxC. We employed isothermal titration calorimetry for the measurement of affinity between AaLpxC mutants and ACHN-975, and the results manifest the key residues (His74, Thr179, Tyr212, His226, Asp230 and His253) for interaction. The determined AaLpxC crystal structure in complex with ACHN-975 is expected to serve as a guidance and basis for the design and optimization of molecular structures of ACHN-975 analogues to develop novel drug candidates against Gram-negative bacteria.

## 1. Introduction

The alarmingly continued rise of bacterial resistance against antibiotics poses a considerable threat to human health in the 21st century [1]. The “ESKAPE” pathogens (*Enterococcus faecium*, *Staphylococcus aureus*, *Klebsiella pneumoniae*, *Acinetobacter baumannii*, *Pseudomonas aeruginosa* and *Enterobacter species*) [2] are responsible for the majority of nosocomial infections. Whereas significant development toward the discovery of novel agents targeting Gram-positive ESKAPE microorganisms has been made positive progress, few, in numerous instances, drugs exist against the multi-drug resistant Gram-negative bacteria [3]. So much of bacterial pathogen threats have triggered the drug research efforts to discover novel antibiotics of increased potency along with expanded spectrum particularly targeting the Gram-negative bacterial pathogens. It is known that Gram-negative bacterial pathogens harbor an extra outer membrane, which consists of lipopolysaccharide (LPS), which is composed of O-antigen, core polysaccharide, and lipid A. LPS offers a remarkable defensive barrier, and the Gram-negative bacterial pathogens that lack lipid A are either highly susceptible or not responsive to a scope of anti-infective medicines, implying that the enzymes responsible for lipid A biosynthesis might be taken as novel therapeutic targets [4]. UDP-3-*O*-(*R*-3-hydroxymyristol)-*N*-acetylglucosamine deacetylase (LpxC), as the critical enzyme in the first non-reversible step for the biosynthesis of lipid A, is a cytosolic zinc-based deacetylase that in no doubt is of particular concern. The significant role that LpxC plays in lipid A biosynthesis along with the advantage of lacking homologous mammalian proteins, makes it an excellent target for the development of small molecular LpxC inhibitors for curing serious Gram-negative infections.

**Figure 1:**
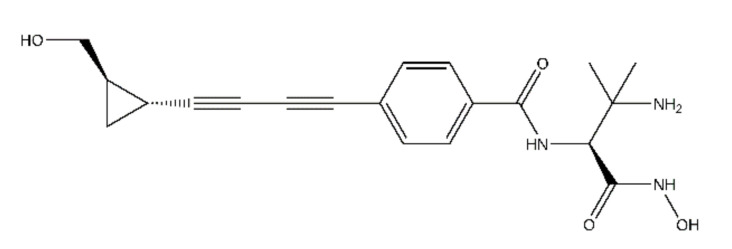
Chemical structural formula of ACHN-975.

Various structural classes of LpxC inhibitors with antimicrobial activity and antibacterial spectrum against Gram-positive pathogens have been reported so far (Figure 1 and Supplementary Figure S1). The substrate analogue inhibitor TU-514 utilizes the zinc-coordination, as well as the fatty-acid binding and *N*-acetylglucosamine binding sites of LpxC [5,6]. It was recently reported that a new biphenylacetylene-based LpxC inhibitor of LPC-069 has activity against MDR/XDR carbapenemase producing strains [7]. Up to date, there have been more than a dozen patent-registered applications for LpxC-targeted compounds, which showed enzyme suppression. Common structural features of these inhibitors are the structural element addressing the UDP binding site or the hydrophobic tunnel. Nevertheless, to our knowledge, none of these patented compounds have been marketed successfully as a drug. Researchers from Achaogen Inc., in 2012 disclosed a series of butadiyne derivatives in patent WO2012154204. Among the hydroxamates identified in this patent, ACHN-975 was shown to be bioactive (MIC ≤ 4 μg/mL) against 98.3% of 120 Gram-negative isolates consisting of *E. coli* ATCC25922, *K. pneumoniae* ATCC43816, and *Y. enterocolitica*. Owing to promising data obtained from the preclinical studies, ACHN-975 constituted the first LpxC inhibitor to enter human clinical trials. However, clinical studies of ACHN-975 were halted because of inflammation at the infusion site [8].

As far as LpxC is concerned, several crystal structures of homogeneous LpxCs from the *Aquifex aeolicus* (AaLpxC) [9], *Escherichia coli* (EcLpxC) [10], *Pseudomonas aeruginosa* (PaLpxC) [11], and *Yersinia enterocolitica* (YeLpxC) [12] have been reported previously. Furthermore, the structure of LpxC in complex with substrate analog TU-514 [6], the N-aroyl-L-threonine derivative CHIR-090 [12,13], the sulfonamide BB-78485 [11], as well as the uridine-based inhibitor 1-68A [14], share the Zn^2+^-binding hydroxamic acid moiety that is bound to a structural element addressing the UDP binding site or the hydrophobic tunnel. Among them, the three-dimensional structure of AaLpxC being explored by X-ray crystallography [9] and NMR spectroscopy [5] revealed a “β-α-α-β sandwich” fold and metal (Fe^2+^ or Zn^2+^)-binding motif. These structural evaluations further showed comprehensive information about the binding mode of substrate molecules and inhibitors [15]. In recent years, the co-crystal structure of AaLpxC complexed with LPC-009 [16], LPC-069 [7], and CHIR-090 [17] have been solved, respectively, reporting the details of inherent structural features of AaLpxC for improving inhibitors as broad-spectrum LpxC-targeting antibiotics [10,16,18]. In particular, small molecules able to inhibit LpxC characterize a novel mode of antibacterial action with a low probability of being ineffective through the pre-existing target specific mechanisms of resistance [19].

To aid in the development of new compounds, which target LpxC, we present a crystallographic assessment on a complex of AaLpxC with ACHN-975, which allows us to elucidate the molecular recognition of ACHN-975 binding site within the enzyme. The obtained crystal structure of LpxC with ACHN-975 is expected to provide guidance and basis for the design and optimization of the small molecules structure of ACHN-975 analogues.

## 2. Results

### 2.1. Overall Structure of AaLpxC/ACHN-975 Complex

**Figure 2:**
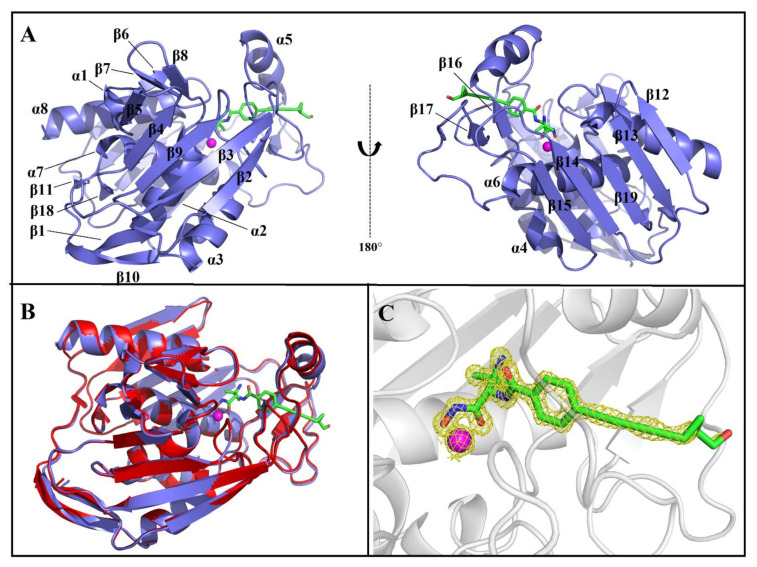
The crystal structure of AaLpxC in complex with ACHN-975. (**A**) Overall structure of LpxC from *A. aeolicus*. The 8 α-helices and 19 β-sheets represent α1–α8 and β1–β19, respectively. (**B**) Superimposition of the overall structures of monomeric AaLpxC in the *apo* form (red, PDB: 1P42) and AaLpxC·ACHN-975 (slate). (**C**) 2Fo–Fc map showing the ACHN-975 and Zn^2+^ calculated at 2.0 σ. The ACHN-975 molecule is shown as sticks. The Zn^2+^-ion is depicted as magenta sphere.

The inhibitor-bound co-crystal structure of the AaLpxC was assessed by using molecular replacement to a resolution at 1.21 Å with final *R*_work_ and *R*_free_ being 16.41% and 18.11%, respectively. Table 1 indicates the data collection, as well as the refinement statistics. In brief, the overall fold of AaLpxC·ACHN-975 constitutes the α+β class and its tertiary structure is generated from two domains linked via a 16-residue linker, with each domain composed of a two α-helices and five-stranded β-sheet with mixed parallel as well as antiparallel orientation, with the active site positioned at the interface of the two domains. The active site is additionally flanked by the small βββ subdomain, βαβ subdomain and Zn^2+^ (Figure 2A). Due to the disorder at the C-terminus, the last five amino acids of AaLpxC (LTRLE) were not able to model into the final structure. The AaLpxC exhibits an extensive rigid structure, which has very limited variation in conformation upon bindings of diverse inhibitors. Similarly, ACHN-975 binding was reported to trigger inconspicuous protein conformational changes. Superimposing the AaLpxC·ACHN-975 complex and its *apo* form (PDB: 1P42) leaves root mean square deviations between corresponding Cα atoms of 0.299 Å. Therefore, the overall structure of the AaLpxC·ACHN-975 complex is essentially identical to that of its *apo* form (Figure 2B). Additionally, seven water molecules enter the binding cavity and form a water-mediated hydrogen bond network of interactions with H19, H58, E73, H74, T179, H226, K227, D230, H253, and ACHN-975 (Supplementary Figure S2). The final structure shows a good overall quality as evidenced by the statistics presented in Table 1.

### 2.2. ACHN-975 Binding

**Figure 3:**
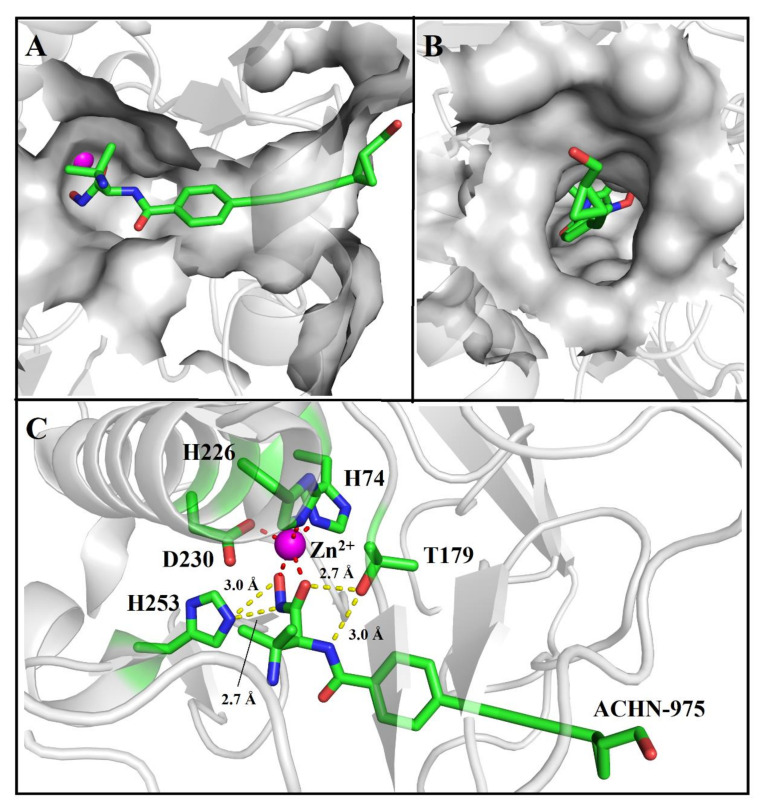
Crystal structure of ACHN-975 attached to AaLpxC. (**A**) The hydroxamic acid moiety of ACHN-975 (green) at the head group of the molecule is shown linking the active site Zn^2+^ ion (magenta sphere) with the tail side of the molecule extending via a hydrophobic tunnel. (**B**) The hydroxymethyl cyclopropyl tail group extrudes completely out of the tunnel exit surface. (**C**) A view of ACHN-975 within the active site. ACHN-975, as well as the side chains of Zn^2+^-binding residues and essential catalytic residues of AaLpxC are indicated as a stick model. The Zn^2+^-ion is depicted as a magenta sphere. Dashed lines illustrate the zinc coordination (red) and hydrogen bond (yellow) cross talks.

**Figure 4:**
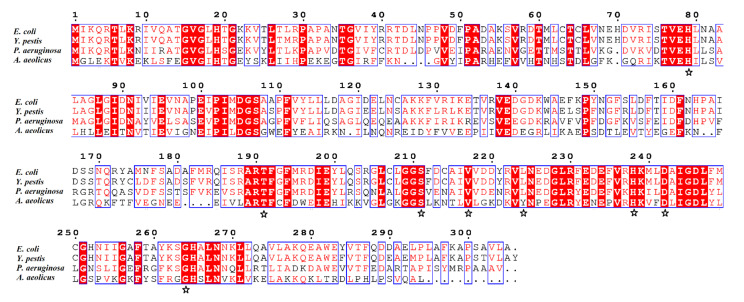
Sequence alignment of diverse LpxC orthologs. The sequences are from *Escherichia coli* (UniProtKB ID: P0A727.1), *Yersinia pestis* (NCBI ID: Q8ZIE3.2), *Pseudomonas aeruginosa* (UniProtKB ID: P47205.2), and *Aquifex aeolicus* (NCBI ID: O67648.1). Residues indicated in red are similar or identical. The key ligand-binding residues are marked with asterisks.

Herein, the crystal structure of the AaLpxC in complex with ACHN-975, provides the structurally binding details at atomic level of ACHN-975 inhibitory mechanism for the first time. We established that the binding site of ACHN-975 is positioned between strand β16, β17, and helix α5 of AaLpxC (Figure 2A). In the AaLpxC·ACHN-975 complex, the Zn^2+^ coordination polyhedron constitutes a five-membered coordination complex with an approximate square pyramidal geometry. The location of the catalytic Zn^2+^ is at the base of the ~20 Å deep, conical active site cleft (Figure 3A,B), which is coordinated by H74 of α-helix 2 and H226, D230 of α-helix 6. A HKΦΦD sequence characterizes this motif in which the residues are conserved throughout the LpxC family (Figure 4). As Figure 3C shows, the ACHN-975 extensively interacts with AalpxC via hydrogen bonds with T179 (2.7 Å and 3.0 Å bond length) and H253 (2.7 Å and 3.0 Å bond length). The hydroxamate moiety of ACHN-975 is within the hydrogen linking distance of the imidazolium side chain of H253. The hydroxamate hydrogen atom is between the hydrogen link distance of H253, which is supposed to be a donor of a hydrogen bond given its increased pKa of ~8. The hydroxamate C=O group of ACHN-975 accepts a hydrogen bond from T179, and NH group of ACHN-975 acts as donor of a hydrogen bond to T179.

### 2.3. Charting the Thermodynamic Parameters of the Complexes via ITC

It is generally accepted that the amino acids that participate in the substrate binding or catalytic sites are crucial for the catalysis or inhibition. The following amino acid residues of AalpxC, E7, I18, H19, S59, H74, T179, P180, E185, I186, I189, S199, L200, V205, Y212, H226, K227, D230, and H253, are selected as “hot spots” (Supplementary Figure S3) to identify putative residues participating in the binding of ACHN-975. ITC was employed to evaluate the thermodynamic parameters of the two complexes. We used ITC and L-alanine scanning mutagenesis to further characterize the interaction between the WT/mutants with ACNH-975. Results (Table 2 and Supplementary Figure S4) showed that H74A, T179A, S199A, V205A, Y212A, H226A, D230A, and H253A had no measurable binding affinity against ACHN-975, which was consistent with the function of these residues as binding sites. Similarly, the binding affinity parameters showed that the *K_d_* values of the T203A mutations were 4.0 times more than that of the WT. In addition, E73A, F108A, E185A, and L200A exhibited increased binding affinity against ACHN-975 compared with the WT, respectively (Table 2).

## 3. Discussion

LpxC exhibits no sequence homology with other zinc-metalloenzymes, as well as any mammalian protein [20], which is conserved in virtually all Gram-negative bacteria [16]. Its orthologs, nonetheless, share significant sequence similarity (Figure 4), with AaLpxC being similar to EcLpxC (33% sequence identity), YeLpxC (32% sequence identity), and PaLpxC (37% sequence identity). Superposition of the four proteins separately shows that the conformation of AaLpxC is similar to EcLpxC (PDB: 3P3G, average r.m.s. deviation of 1.087 Å), YeLpxC (PDB: 3NZK, average r.m.s. deviation of 0.752 Å), and PaLpxC (PDB: 4LCF, average r.m.s. deviation of 0.817 Å). Despite the relative low sequence similarity of AaLpxC to its orthologs from *E. coli*, *Y. pestis*, as well as *P. aeruginosa*, their three-dimensional structures are quite well conserved. This should allow the design of new antimicobial agents that are selective against Gram-negative bacterial pathogens with limited off-target effects [21]. The ACHN-975 was docked with the EcLpxC and YeLpxC using Autodock Vina, respectively (Supplementary Figure S5). Interestingly, superimposition of EcLpxC/ACHN-975 and YeLpxC/ACHN-975 with AaLpxC/ACHN-975 structures show very limited conformational variation. The results are consistent with antibacterial activity of ACHN-975 against E. coli and Y. enterocolitica. The structures of AaLpxC, as well as other LpxC orthologues in complex with numerous LpxC inhibitors, have been determined through NMR spectroscopy along with X-ray crystallography [9,13,22]. It is well known from previous studies that inhibitors of LpxC, which showed antibacterial capacity against Gram-negative bacteria, have shared two structural elements, namely (1) a hydroxamic acid moiety as metal binding unit [10]; (2) a rigid and linear lipophilic tail to fill the hydrophobic tunnel [10,16,18]. Several LpxC inhibitor researches yielded a number of prospective lead molecules, however, only ACHN-975 reached human clinical trial. We believe that our results as well as the analysis herein complement and enrich the structurally binding mechanism on AaLpxC-inhibitor interaction.

**Figure 5:**
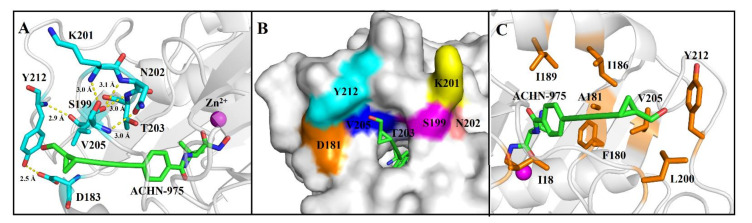
Close-up views of ACHN-975 with surrounding residues. (**A**) The models of the amino acids of S199, Y212 and their surroundings. The dotted lines indicate a hydrogen-bonding network around the residues at the position 199 and 212. (**B**) A view of the ACHN-975 binding onto AaLpxC. Highlighted in surface view are amino acids involved in shaping the substrate pocket. ACHN-975 (white), D181 (oranges), S199 (magentas), K201 (yellow), N202 (salmon), T203 (lightblue), and V205 (blue). (**C**) Close-up views of ACHN-975 with surrounding aliphatic residues. The ACHN-975 and surrounding residues are indicated as stick models. The Zn^2+^-ion is depicted as a magenta sphere.

The AaLpxC·ACHN-975 complex X-ray structures yield structure and interaction information on inhibitor docking in the active site. The enzyme’s van der Waals surface exhibits a ~20 Å deep active-site cleft at the base of which is a Zn^2+^ in the AaLpxC (Figure 3A). The five-valenced coordination Zn^2+^ is formed by H74, H226, D230, and ACHN-975 (Figure 3C). Prior Site-directed mutagenesis studies have identified the H74, H226, and D230 of AaLpxC and EcLpxC (H79, H238 and D242) to be the conserved residues to coordinate the Zn^2+^-ion [23]. We performed calorimetric titrations of ACHN-975 with these H74A, H226A, and D230A mutants, but we were unable to detect any interaction, which implied that these residues are key residues of substrate binding. Site-targeted mutagenesis of the conserved His, and Asp residues of AaLpxC, identified H74 and H226 as probable ligands of the catalytic Zn^2+^-ion and opined D230 to be the third side chain to coordinate the Zn^2+^-ion [23]. AaLpxC interacts with ACHN-975 via hydrogen bonding interactions on T179 site. T179A mutation led to the predicted loss of its hydrogen link with the amine-containing head group of ACHN-975, indicating abolish ACHN-975 binding. Compared to the structures of the AaLpxC·LPC-004 complex, the corresponding benzolactam derivative exhibited a 100-fold lower potency [24], revealing the significance of the hydrogen bond between the amide NH of LPC-004 and T179. Similarly, the research of AaLpxC·CHIR-090 complex provided a coordinated relationship between the hydroxamate moiety of CHIR-090 with Zn^2+^-ion and a key hydrogen bond between the amide proton of its threonyl group with the hydroxyl group of T179. Taken together, these studies demonstrate that the hydroxamic acid group is a core feature, which accounts for the high affinity binding of the inhibitor to AaLpxC. Furthermore, a separate ~15 Å long hydrophobic tunnel similarly leads to the Zn^2+^ (Figure 3B). This hydrophobic tunnel, being formed by several aliphatic residues (I18, I186, I189, L200, and V205), leads out of the active site and hosts the hydroxymethyl cyclopropyl tail group of ACHN-975. The aliphatic tail of ACHN-975 protrudes into a groove between strand β16, β17, and helix α5, which has been suggested to be the binding site for the substrate/inhibitor. S199A and Y212A are not affinity binders to ACHN-975, which we characterized by ITC with the *K_d_* value (Table 2). S199 and Y212, another vital binding site, have been documented in the AaLpxC crystal structures. Point mutations Y212A was shown to block polar interactions with hydroxymethyl of ACHN-975 and hydrogen bonding interactions with carboxyl group of D183 (Figure 5A). It is interesting to note that Y212 is not a conservative amino acid, as this site of *E. coli*, *Y. pestis* and *P. aeruginosa* is leucine, which has no interaction with ACHN-975 [25]. The hydrogen bonds in S199 with K201, N202, and T203 give rise to hydrogen bonding networks that have a core role of maintaining the shape of the substrate binding pocket (Figure 5B). Mutation of serine to alanine (S199A) indeed abolished the affinity activity of AaLpxC. These residues, S199 and Y212, were considered the key residues to stabilizing and orienting the substrates for the binding. By contrast, the single amino-acid mutation I18A, H19A, S59A, I189A, and K227A had a slight effect on affinity compared to the WT. Intriguingly, ITC measurement demonstrated that the binding cross talk is spontaneous in approach and the electrostatic forces along with hydrogen bonding constitute the predominant binding forces (Supplementary Figure S4).

Here we have described the interactions of AaLpxC with the small molecule hydroxamate ACHN-975, which may function as prospective new antibacterial agents for treating the Gram-negative bacterial infections. There is an effort to improve the antimicrobial activity of ACHN-975 against other Gram-negative bacteria, to widen the therapeutic window and to optimize its physicochemical parameters. We compared the structures of AaLpxC/ACHN-975 with AaLpxC/LPC-009 and AaLpxC/CHIR-090. Both include the five-valenced coordination Zn^2+^ that is formed by H74, H226, D230, and the inhibitor in these structures. Several conserved hydrophobic amino acids (I18, F180, I186, I189, L200, and V205) were expected to engage the hydrophobic channel (Supplementary Figure S6). Interestingly, there were more hydrophobic interactions with surrounding residues in the structure of AaLpxC/CHIR-090 compared with the AaLpxC/ACHN-975 and AaLpxC/LPC-009. Undoubtedly, the structure of diphenylacetylene linker has played a key role in hydrophobic interaction. Here we present some recommendations that may be useful to medicinal chemists in future structural optimization of ACHN-975 (Supplementary Figure S7). (i) The primary amino group could be connected with a water-soluble group that could occupy the UDP pocket, to increase the binding affinity. (ii) The benzamide bis-acetylene linker could be replaced by diphenylacetylene linker, since the hydrophobic tunnel of AaLpxC has sufficient space to accommodate the benzene ring; in this case, the ACHN-975 derivative may form additional hydrophobic interactions with amino acid residues in the hydrophobic tunnel. (iii) The cyclopropyl group could be replaced by heterocycles containing nitrogen atoms, such as pyridine, pyrimidine, morpholine, and pyrrolidine, in further structural modifications, in order to find novel ligands with better binding affinity and structural diversity. Our structural and functional studies of AaLpxC·ACHN-975 provide the framework to elucidate the molecular basis of inhibitory and binding mechanism. These intermolecular cross talks provide a basis for comprehending the structural aspects on the enzyme-substrate, as well as enzyme-inhibitor affinity.

## 4. Materials and Methods

### 4.1. Cloning and Site-Directed Mutagenesis of AaLpxC

The *aalpxc* gene of AaLpxC (accession number: WP_010881152.1) from *Aquifex aeolicus* with codons optimized to permit expression in the suitable *Escherichia coli*, was PCR-amplified by Pyrobest™ DNA Polymerase (TaKaRa) with a set of primers AaLpxC-F: 5′- GGAATTCCATATGGGCCTGGAAAAAACCGTGAAGG-3′, and AaLpxC-R: 5′- CCGCTCGAGGCGGGTCAGTTTCTGTTTTTTAGCC-3′, which contained an 5′-terminal Nde I and a 3′-terminal Xho I restriction sites (underlined), respectively. The *aalpxc* insert was consequently ligated into NdeI-, as well as XhoI-digested pET-21a (Novagen). The integrity of this construct, pET21a-AaLpxC, was verified by DNA sequencing. Site-directed mutagenesis on amino acids in the *aalpxc* gene was attained via overlapping extension PCR to generate recombined pET21a-mutants plasmid. Following the confirmation of the sequences, these plasmids containing modified *lpxc* gene were transformed separately into the competent *E. coli* BL21 (DE3) for heterologous expression. The Supplementary Table S1 indicates all the oligonucleotide primers.

### 4.2. Protein Expression and Purification

We utilized a single colony to inoculate into 10 mL of LB medium enriched with ampicillinum (100 mg L^−1^) and it was overnight-incubated at 310 K. Afterwards, dilution of the culture (1:100) into 1 L fresh medium containing ampicillinum (100 mg L^−1^) was done and incubated at 310 K until the culture attained an OD_600_ of between 0.6 and 0.8. Thereafter, 0.1 mM of isopropyl β-D-thiogalactopyranoside was employed to stimulate expression at 293 K and the medium was enriched with 0.5 mM-sterile ZnSO_4_ at this point. About 16 h later, we harvested the cells through spinning at 277 K for 15 min at 6000 g and then kept at 253 K.

We re-suspended the pelleted cells in the lysis buffer containing 10 mM imidazole, 20 mM HEPES (pH 7.5), as well as 300 mM NaCl, and then disrupted the, by a high-pressure homogenizer. The soluble fraction was centrifuged at 48,400× *g* for 40 min at 277 K, and then filtration via a 0.45 mm filter was done and subsequent loading onto a Zn^2+^-NTA agarose gel column (Qiagen) equilibrated using the lysis buffer. Afterwards, protein elution through a linear imidazole gradient (20–500 mM) was done in the same lysis buffer. The purity along with the molecular weight of the proteins were verified using a 10% SDS-PAGE and the fractions with pure targeted protein were pooled, and dialyzation against 20 mM Tris-HCl (pH 8.0) and 10 mM NaCl was performed. Then, we applied the sample onto a 5 mL HiTrapTM Q HP column (GE Healthcare, Chicago, IL, USA). Afterwards, a linear gradient of NaCl (20 to 500 mM) was employed in eluting the protein. Pooling of fractions that contained LpxC was conducted and further purification through gel-filtration chromatography (Superose 12 10/300 GL, GE Healthcare) performed in 20 mM HEPES (pH 7.5), 100 mM KCl. Purity was ascertained via SDS–PAGE and the target protein has a molecular weight of approximately 32 kDa consistent with the expected AaLpxC size as indicated in the Supplementary Figure S1.

### 4.3. Crystallization and Data Collection

Initial screenings were performed with Crystal Screen 2 HR2-112 and HR2-110 (Hampton Research, Aliso Viejo, CA, USA), as well as Wizard CRYOI, Wizard CRYOII (Rigaku, Austin, TX, USA) via the sitting drop format of vapor diffusion at 20 °C, then, positive hits were optimized. After optimizing the conditions, the AaLpxC crystals were grown through the hanging-drop vapor diffusion at 288 K in 1 μL protein solution (0.25 mM), as well as 1 μL of a reservoir solution (100 mM HEPES pH 7.0, 6% (*w*/*v*) PEG8000, 10% (*v*/*v*) glycol) supplemented with 5 mM ACHN-975. Flash-cooling of the crystals was done to a temperature of~100 K by liquid nitrogen with cryo-protectant containing 100 mM HEPES, pH 7.0, 6% (*w*/*v*) PEG8000, and 10% (*v*/*v*) glycol. Collection of all the X-ray diffraction data was performed at the Shanghai Synchrotron Radiation Facility (SSRF) on beam line BL17U1 with a CCD detector with a beam at wavelength of 0.97915 Å [26].

### 4.4. Structural Determination and Refinement

Data processing was performed with the HKL2000 software package [27]. Crystals of AaLpxC·ACHN-975 diffracted the X-rays to 1.21 Å resolution. The molecular replacement approach with an existing ligand-free structure (Protein Data Bank (PDB) code: 1P42) [9] as research model was employed to solve the structure. The program COOT [28] was employed to adjust the structure and consequently the program Phenix.refine [29], with excellent stereochemistry being employed to refine the structure. The placement of ACHN-975 was finished by the LigandFit within Phenix, and the final refinement was done by adding water molecules with final *R_work_* and *R_free_* being 16.41% and 18.11%, respectively. The last five residues at the C-terminus are absent. Structural figures were drawn using the PyMOL software (The PyMOL Molecular Graphics System, V. 1.3, Schrödinger, LLC, New York, USA). Clustal X [30] was employed to perform sequence alignments and ESPript 3.0 [31] utilized to generate the final output.

### 4.5. Isothermal Titration Calorimetry

The Malvern-MicroCal PEAQ-ITC Microcalorimeter system was employed to perform the ITC experiments. We connected this device to a computer with MicroCal PEAQ-ITC software for controlling the device as well as to record the data. Prior to all the experiments, we loaded 200 μL of 30 μM AaLpxC solution to the sample cell, and then loaded 150 μM of ACHN-975 solution to the injection syringe. Post the equilibration time of the calorimeter, there was a 60 s delay prior to titration. We set the stirring speed to 800 rpm. Each experiment was conducted at least in duplicate under the temperature setting of 25 °C; the reference power was set at 5 μcal/s; the injection volume was set at 2 μL as first injection and then followed by 15 μL for the progressive 19 injections; with a 200 s spacing between injections. The Origin 7 software provided by the manufacturer with curves fitted with one set of site models was employed to analyze the data.

## Figures and Tables

**Table 1:** X-Ray Data Collection and Structure Refinement Statistics.

Data Collection	AaLpxC·ACHN-975
Space group	P6_1_
Cell dimensions	
a, b, c (Å)	65.569, 65.569, 131.595
α, β, γ (°)	90.000, 90.000, 120.000
Resolution (Å)	50–1.21 (1.25–1.21)
*R*_merge_	0.084 (0.600)
*I/*ϭ	35.0 (5.4)
Completeness (%)	95.3 (91.0)
Redundancy	19.6 (19.9)
Refinement	
Resolution range	50−1.21 (1.25–1.21)
No. of reflections	92652 (8841)
Redundancy	19.6 (19.9)
*R*_work_/*R*_free_	0.1641/0.1811
No. of atoms	
Protein	2157
Ligand/ion	45
Water	350
Average *B*-factors	
Protein	19.35
Ligand	22.56
Zn^2+^	26.27
Root mean square deviations	
Bond lengths (Å)	0.006
Bond angles (°)	1.036
Ramachandran	
Favored (%)	96.23
Allowed (%)Outliers (%)	90.000.00
Protein Data Bank Code	6IH0

Values in parentheses are for the highest resolution shell. *R_merge_* = Σ*_hkl_* Σ*_i_* |*I_i_*(*hkl*) − <*I*(*hkl*)>|/Σ*hkl* Σ*_i_* *I_i_*(*hkl*), where *Ii*(*hkl*) is the intensity of the *i*th observation of reflection *hkl* and <*I*(*hkl*)> is the average intensity of reflection *hkl*. *R*_free_ was calculated with 5% of all reflections excluded from refinement stages using high resolution data.

**Table 2:** Determination of affinity between AalpxC and mutants with ACHN-975.

Mutant	*K*_d_ (μM)	∆H (kcal/mol)	∆G (kcal/mol)	−T∆S (kcal/mol)
WT	0.4 ± 0.1	−8.2 ± 0.5	−8.8	−0.6
I18A	0.4 ± 0.3	−6.6 ± 0.9	−8.8	−2.2
H19A	0.4 ± 0.2	−4.5 ± 0.4	−8.8	−4.3
S59A	0.4 ± 0.1	−9.1 ± 0.4	−8.7	0.38
E73A	0.07 ± 0.05	−7.4 ± 0.5	−9.7	−2.4
H74A	NA	NA	NA	NA
T179A	NA	NA	NA	NA
F180A	0.09 ± 0.07	−2.8 ± 0.2	−9.6	−6.8
E185A	0.06 ± 0.03	−8.1 ± 0.3	−9.9	−1.8
I186A	0.2 ± 0.09	−5.4 ± 0.40	−9.3	−3.9
I189A	0.1 ± 0.09	−7.0 ± 0.6	−9.4	−2.4
S199A	NA	NA	NA	NA
L200A	0.07 ± 0.02	−4.8 ± 0.3	−11.1	−6.3
T203A	2.0 ± 1.4	−7.0 ± 1.6	−7.8	−0.8
V205A	NA	NA	NA	NA
Y212A	NA	NA	NA	NA
H226A	NA	NA	NA	NA
D230A	NA	NA	NA	NA
K227A	0.1 ± 0.08	−6.0 ± 0.49	−9.5	−3.4
H253A	NA	NA	NA	NA

Values obtained from 2–3 repeated ITC experiments. The stoichiometry (AaLpxC: ACHN-975, *n*) = 0.23–0.72.

## Data Availability

Not available.

## Supplementary Materials

The following are available online. Figure S1: Representative LpxC inhibitors, Figure S2: The water-mediated hydrogen bond network of interactions with surrounding residues, Figure S3: SDS-PAGE analysis of purified recombinant AaLpxC and mutants, Figure S4: ITC raw data and binding isotherm for ACHN-975 interacting with the AaLpxC (WT) and the mutants, Figure S5: Superimposition of EcLpxC/ACHN-975 and YeLpxC/ACHN-975 with AaLpxC/ACHN-975 structures, Figure S6: Structural comparison of AaLpxC/ACHN-975 with AaLpxC/LPC-009 and AaLpxC/CHIR-090, Figure S7. Schematic of recommended ACHN-975 derivatives; Table S1: Primers for site-directed mutagenesis.
