# Supplementary material for: The Complex Structure of Protein AaLpxC from Aquifex aeolicus with ACHN-975 Molecule Suggests an Inhibitory Mechanism at Atomic-Level against Gram-Negative Bacteria
Source: Molecules. 2021 Mar 7;26(5):1451. doi: 10.3390/molecules26051451 (PMC7962117; doi:10.3390/molecules26051451)
Supplement: Supplementary file 1 [file molecules-26-01451-s001.pdf]

Supplementary Table S1 Primers for site-directed mutagenesis. Point mutations are underlined.

Mutant	Name	Sequence (5′ → 3')
I18A	I18A-F	AAGGTGTGGGCGCACACACCGGCGA
	I18A-R	TCGCCGGTGTGTGCGCCCACACCTT
H19A	H19A-F	GGTGTGGGCATTGCAACCGGCGAGT
	H19A-R	GTACTCGCCGGTTGCAATGCCCACA
S59A	S59A-F	CATACCAACCACGCAACAGATCTGG
	S59A-R	CCCAGATCTGTTGCGTGGTTGGTAT
E73A	E73A-F	TATCAAAACCGTGGCGCATATCCTG
	E73A-R	GCTCAGGATATGCGCCACGGTTTTG
H74A	H74A-F	AAACCGTGGAGGCAATCCTGAGCGT
	H74A-R	GCTCAGGATTGCCTCCACGGTTTTG
T179A	T179A-F	TGCTGGCCCGCGCATTCGCCTTCGA
	T179A-R	AATCGAAGGCGAATGCGCGGGCCAG
F180A	F180A-F	TGGCCCGCACCGCAGCCTTCGATTG
	F180A-R	CAATCGAAGGCTGCGGTGCGGGCCA
E185A	E185A-F	GCCTTCGATTGGGCAATTGAACATA
	E185A-R	GATATGTTCAATTGCCCAATCGAAG
I186A	I186A-F	TCGATTGGGAAGCAGAACATATCAA
	I186A-R	TGATATGTTCTGCTTCCCAATCGAA
I189A	I189A-F	GAAATTGAACATGCAAAGAAAGTGG
	I189A-R	CACTTTCTTTGCATGTTCAATTTCC
S199A	S199A-F	AAAGGCGGTGCACTGAAGAACACCC
	S199A-R	GGTGTTCTTCAGTGCACCGCCTTTG
L200A	L200A-F	AGGCGGTAGCGCAAAGAACACCCTG
	L200A-R	CAGGGTGTTCTTTGCGCTACCGCCT
T203A	T203A-F	CCTGAAGAACGCACTGGTGCTGGGT
	T203A-R	ACCCAGCACCAGTGCGTTCTTCAGG
V205A	V205A-F	GAACACCCTGGCACTGGGTAAAGAC
	V205A-R	TCTTTACCCAGTGCCAGGGTGTTCT
Y212A	Y212A-F	AAGACAAAGTGGCAAATCCGGAAGG
	Y212A-R	GCCTTCCGGATTTGCCACTTTGTCT
H226A	H226A-F	CCGGTTCGCGCAAAGGTTTTCGACC
	H226A-R	GGTCGAAAACCTTTGCGCGAACCGG
K227A	K227A-F	CCGGTTCGCCACGCAGTTTTCGACC
	K227A-R	CAGGTCGAAAACTGCGTGGCGAACC
D230A	D230A-F	ACAAGGTTTTCGCACTGATCGGTGA
	D230A-R	GGTCACCGATCAGTGCGAAAACCTTG
H253A	H253A-F	TCCGCGGCGGTGCAAGCCTGAACGT
	H253A-R	ACGTTCAGGCTTGCACCGCCGCGGA

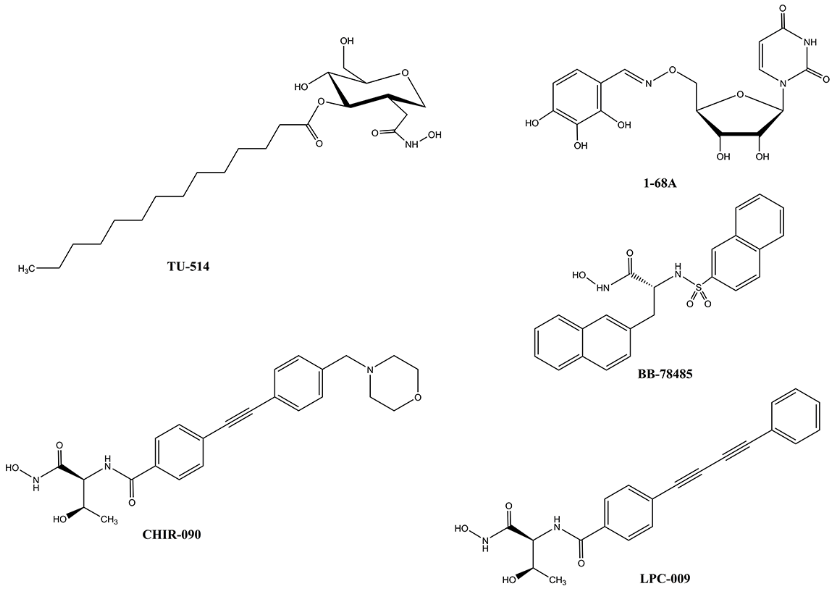

Supplementary Figure S1. Representative LpxC inhibitors

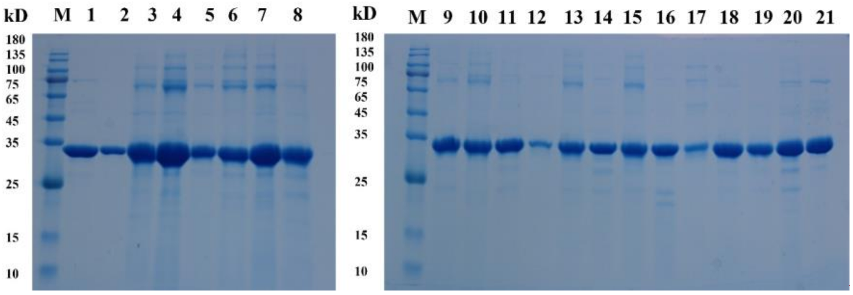

Supplementary Figure S1. SDS PAGE analysis of purified recombinant AaLpxC and mutants. (M: Marker; 1: WT; 2: H19A; 3: T79A; 4: E73A; 5: H74A; 6: H226A; 7: D230A; 8: K227A; 9: WT; 10: I18A; 11: S59A; 12: F180A; 13: E185A; 14: I186A; 15: I189A; 16: S199A; 17: L200A; 18: T203A; 19: V205A; 20: Y212A; 21: H253A)

Formatted: Font: (Asian) +Body Asian (等线)

Formatted: Hyphenate, Allow hanging punctuation

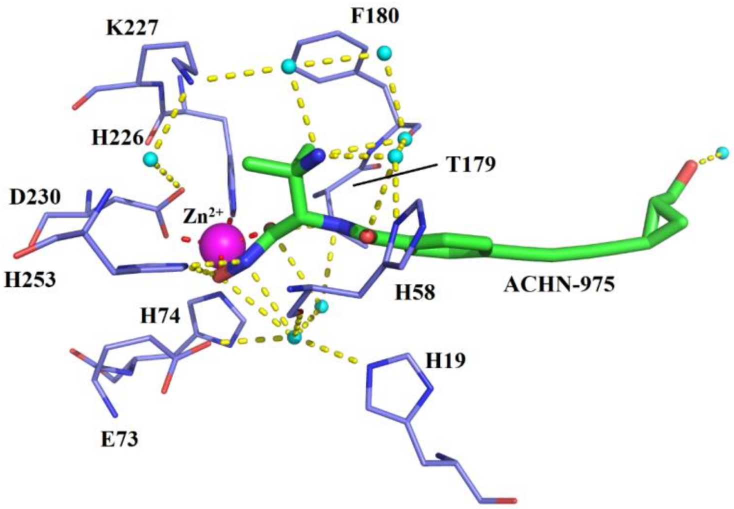

Supplementary Figure S2. The water-mediated hydrogen bond network of interactions with surrounding residues. The surrounding key amino acid residues are labeled as line model, and ACHN-975 is shown as stick model. The Zn^{2+} ion and water molecules are depicted as magenta sphere and cyan sphere, respectively. Dashed lines illustrate hydrogen bond (yellow) cross talks.

Formatted: Font: Not Bold

Formatted: Font: Not Bold, Superscript

Formatted: Font: Not Bold

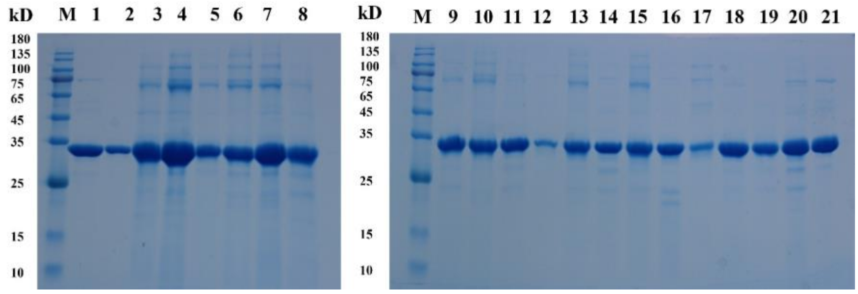

Supplementary Figure S3. SDS-PAGE analysis of purified recombinant AaLpxC and mutants. (M: Marker; 1: WT; 2: H19A; 3: T79A; 4: E73A; 5: H74A; 6: H226A; 7: D230A; 8: K227A; 9: WT; 10: I18A; 11: S59A; 12: F180A; 13: E185A; 14: I186A; 15: I189A; 16: S199A; 17: L200A; 18: T203A: 19: V205A; 20: Y212A; 21: H253A)

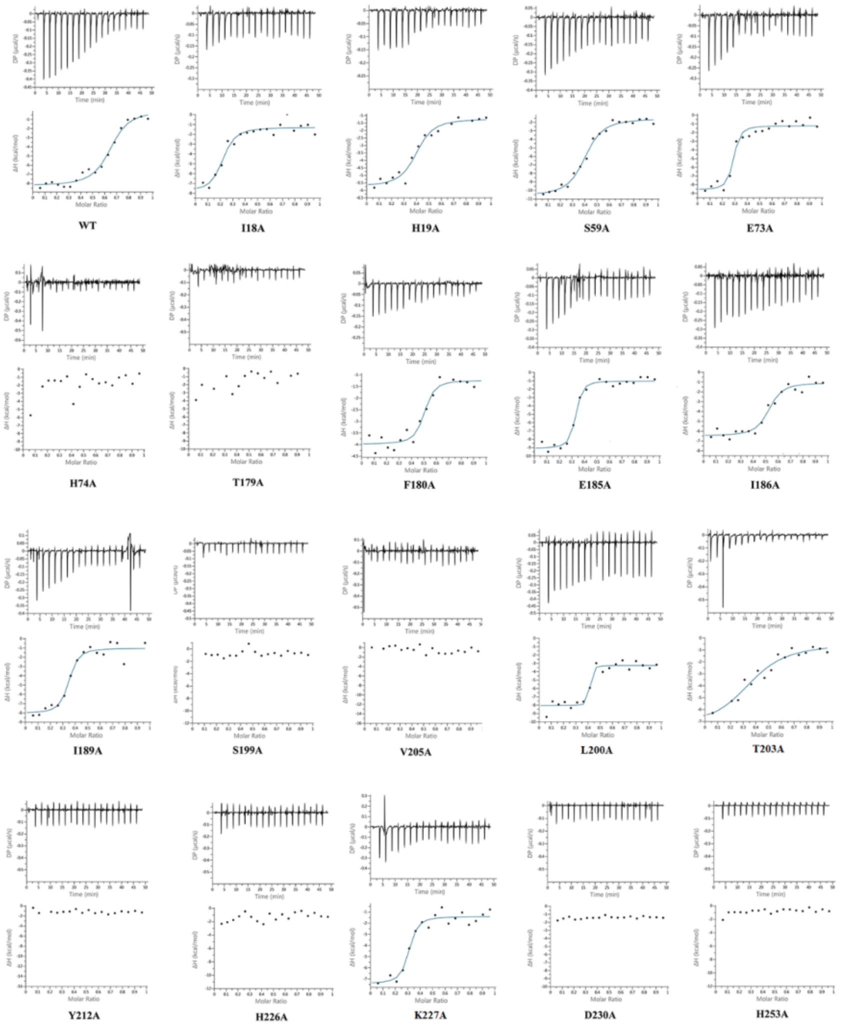

Supplementary Figure ~~S2~~S4. ITC raw data and binding isotherm for ACHN-975 interacting with the AaLpxC (WT) and the mutants. The curves in the figure were obtained from the one-set model using the nonlinear fitting algorithm.

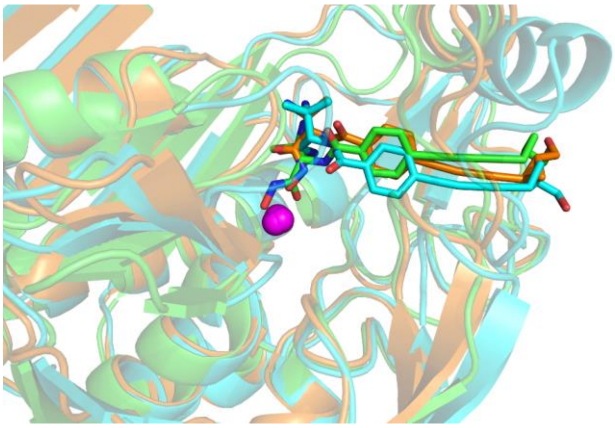

Supplementary Figure S5. Superimposition of EcLpxC/ACHN-975 and YeLpxC/ACHN-975 with AaLpxC/ACHN-975 structures. AaLpxC/ACHN-975, EcLpxC/ACHN-975 and YeLpxC/ACHN-975 are showed in green, cyan and orange, respectively. The ACHN-975 is shown as stick model. The Zn^{2+}-ion is depicted as magenta sphere.

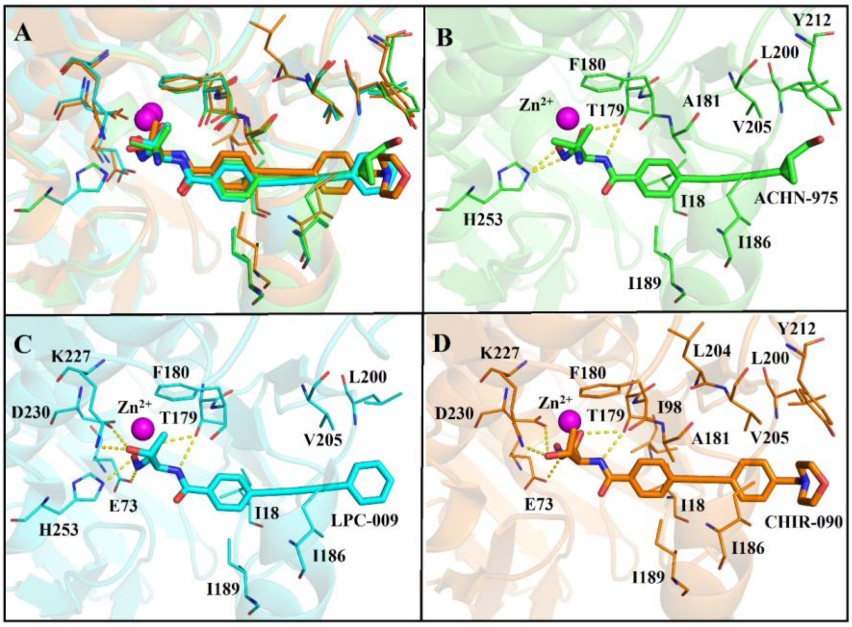

Supplementary Figure S6. Structural comparison of AaLpxC/ACHN-975 with AaLpxC/LPC-009 and AaLpxC/CHIR-090. (A) Superimposition of AaLpxC/LPC-009 and AaLpxC/CHIR-090 with AaLpxC/ACHN-975 structures; (B-D) Detailed view of the AaLpxC/ACHN-975, AaLpxC/LPC-009 and AaLpxC/CHIR-090 binding cavity, respectively. AaLpxC/ACHN-975, AaLpxC/LPC-009 and AaLpxC/CHIR-090 are showed in green, cyan and orange, respectively. The ACHN-975 is shown as stick model. The Zn^{2+}-ion is depicted as magenta sphere. Dashed lines illustrate hydrogen bond (yellow) cross talks.

Formatted: Font: Not Bold

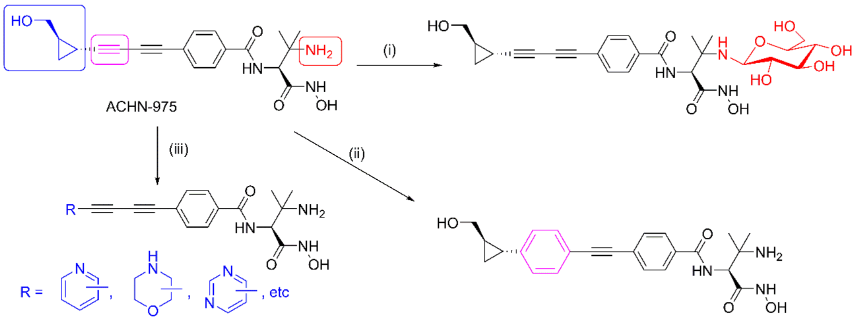

Supplementary Figure S7. Schematic of recommended ACHN-975 derivatives.
